# Age-Related Changes in Bone-Marrow Mesenchymal Stem Cells

**DOI:** 10.3390/cells10061273

**Published:** 2021-05-21

**Authors:** Valentina A. Babenko, Denis N. Silachev, Tatyana I. Danilina, Kirill V. Goryunov, Irina B. Pevzner, Ljubava D. Zorova, Vasily A. Popkov, Valery P. Chernikov, Egor Y. Plotnikov, Gennady T. Sukhikh, Dmitry B. Zorov

**Affiliations:** 1 A.N. Belozersky Institute of Physico-Chemical Biology, Lomonosov Moscow State University, 119992 Moscow, Russia; nucleus-90@yandex.ru (V.A.B.); silachevdn@belozersky.msu.ru (D.N.S.); danilina.tatyana2010@yandex.ru (T.I.D.); irinapevzner@mail.ru (I.B.P.); lju_2003@list.ru (L.D.Z.); popkov.vas@gmail.com (V.A.P.); 2 V.I. Kulakov National Medical Research Center of Obstetrics, Gynecology and Perinatology, 117997 Moscow, Russia; kirishgor@gmail.com (K.V.G.); gtsukhikh@mail.ru (G.T.S.); 3 Research Institute of Human Morphology, 117418 Moscow, Russia; 1200555@mail.ru

**Keywords:** mitochondria, aging, senescence, stem, mesenchymal stromal cells, proliferation, glycolysis, oxidative phosphorylation, glucose utilization, traumatic brain injury, therapy

## Abstract

The use of stem cells is part of a strategy for the treatment of a large number of diseases. However, the source of the original stem cells for use is extremely important and determines their therapeutic potential. Mesenchymal stromal cells (MSC) have proven their therapeutic effectiveness when used in a number of pathological models. However, it remains an open question whether the chronological age of the donor organism affects the effectiveness of the use of MSC. The asymmetric division of stem cells, the result of which is some residential stem cells acquiring a non-senile phenotype, means that stem cells possess an intrinsic ability to preserve juvenile characteristics, implying an absence or at least remarkable retardation of senescence in stem cells. To test whether residential MSC senesce, we evaluated the physiological changes in the MSC from old rats, with a further comparison of the neuroprotective properties of MSC from young and old animals in a model of traumatic brain injury. We found that, while the effect of administration of MSC on lesion volume was minimal, functional recovery was remarkable, with the highest effect assigned to fetal cells; the lowest effect was recorded for cells isolated from adult rats and postnatal cells, having intermediate potency. MSC from the young rats were characterized by a faster growth than adult MSC, correlating with levels of proliferating cell nuclear antigen (PCNA). However, there were no differences in respiratory activity of MSC from young and old rats, but young cells showed much higher glucose utilization than old ones. Autophagy flux was almost the same in both types of cells, but there were remarkable ultrastructural differences in old and young cells.

## 1. Introduction

The assessment of the aging process is mostly quite subjective and there is no unambiguous criterion that would allow us to evaluate the system as senile. However, with some limitations, although not fully defined, aging can be considered as a complex process of gradual decline and loss of essential functions of cells, organs and entire organisms, including the ability to reproduce and regenerate. Due to this, aging is one of the most significant risk factors for most diseases [1]. Age-related increased vulnerability to death urges the entire scientific community to cope with this problem; however, with quite modest success, possibly because of its multifaceted origin and manifestation. Conventionally, the term “aging” is more applicable to an organism, while the decay of cellular functioning is more consistent with the term “senescence”; that said, there is no doubt that cellular aging (senescence) mainly contributes to organismal aging. Some hallmarks of aging on cellular levels were collected and presented as next signs: genomic instability, telomere attrition, epigenetic alterations, loss of proteostasis, deregulated nutrient-sensing, mitochondrial dysfunction, stem cell exhaustion and altered intercellular communication [2].

Possibly, changes associated with aging can affect all cells of the organism, including stem cells; however, the latter may have some specific features regarding aging. The asymmetry of division, which is probably inherent to all dividing cells, is especially pronounced in stem cells in which the best properties (for example, concerning the best quality of inherited mitochondria) are given to those stem cells that remain in the pool, and this determines their stemness [3], while those subjected to differentiation do not have the best properties. It implies that stem cells staying in the pool are more reluctant to senescence and they may not follow the aging process of the entire organism, remaining largely juvenile [4]. Maybe due to this, stem cells play an essential role in organismal homeostasis, and their aging, being possibly retarded, can affect the development of pathologies [5]. The current therapeutical paradigm in regenerative medicine and cell technologies includes the systemic administration of various types of stem and progenitor cells, the main ones of which are multipotent mesenchymal stromal cells (MSC). MSC, mostly isolated from bone marrow, were proposed to be used for the treatment of age-related diseases (heart attacks, strokes, neurodegenerative diseases) [6,7,8,9] or to slow/reverse the aging process [10]. When determining the source of MSC for therapy, two fundamentally different approaches are considered: the use of donor (allogeneic) cells or one’s own (autologous) cells. There are data that stem cells, as well as somatic cells, undergo some chronological deterioration during senescence [11,12,13]. For example, MSC obtained from the same tissue at different ages may differ in the pattern of gene expression [14,15], despite the fact that they have the same phenotype along “classic” surface markers. Accordingly, we face the question: might time affect stem cells and their therapeutic efficacy? On the one hand, there are a sufficient number of preclinical studies demonstrating the therapeutic potential of MSC taken from young sexually mature animals. However, there are practically no data on the effectiveness of cell therapy applied to old individuals, or on the potential of MSC isolated from an old organism. If we consider the introduction of various stem cells into the organism (MSC, hematopoietic, endothelial progenitors, etc.), then it should be noted that there is a dispute in this field as to whether it is acceptable to set a cut-off for donors by biological age [16]. The opinions of experts on this issue diverge and, to date, there is no widely accepted scientific rationale. This raises the question: since physiological aging is a complex process characterized by a progressive decline in organismal integrity, secondary to impaired cellular function, does a progressive decline in MSC occur with age and do MSC from elderly people have noticeable therapeutic potential? First of all, the solution to the problem needs a clear understanding of the aging process as such, while, as we have already mentioned, the level of knowledge of this problem is still rudimentary [17]. However, a set of accumulated data, obtained mainly in vitro, points to functional changes in stem/progenitor cells in aged donors (see for a review [11,14]). It is mostly accepted that aging is associated with changes in the functioning of various cell organelles, including mitochondria, which are one of the key intracellular players involved in the development of aging processes [4]. However, there are no convincing data on the change in energy metabolism in MSC with age and the possible effect of aging on the therapeutic potential of MSC. The aim of this study was to evaluate the physiological changes in MSC from old rats and to compare the neuroprotective properties of MSC from young and old animals in a model of traumatic brain injury (TBI).

The motivation for using of bone marrow MSC in our study was that, initially, bone marrow was the most commonly used source of MSC. MSC are multipotent in nature and can produce tissue of mesodermal lineage [18]. Tissue can be harvested autologously and does not cause ethical, tumorigenic, or immunogenic problems, as presented by pluripotent stem cells. Additionally, the study of bone marrow MSC aging is important in the context of understanding age-related changes in the bone marrow stem cell niche, which represents a population of different interacting cells.

## 2. Materials and Methods

### 2.1. Animals

In experiments, males of outbred Wistar rats (350–400 g) were used. The animals were kept in a temperature-controlled environment (20 ± 2 °C) under natural light/dark cycles with a light on from 9 a.m. to 9 p.m. and free access to standard granular feed and water. Rats were treated according to animal protocols evaluated and approved by the animal ethics committee of A.N. Belozersky Institute of Physico-Chemical Biology: Protocol 3/19 from 18 March 2019. All procedures were in accordance with the Federation of Laboratory Animal Science Associations (FELASA) guidelines.

### 2.2. Rat’s MSC Isolation and Culturing

MSC were isolated from the rat bone marrow of tubular bones of fetuses at 18–19 days of gestation (fetal, fMSC), 7-day rat pups (perinatal, pMSC) and 20–23 months old rats (adult, aMSC). The animals were euthanized by isoflurane, femoral and tibial bones were isolated under aseptic conditions and the cells were washed with Dulbecco’s Modified Eagle Medium (DMEM)/F12 (Paneco, Moscow, Russia) (1:1). Then, the cells were resuspended in complete medium DMEM/F-12 (1:1) containing 7% fetal bovine serum (HyClone, Logan, UT, USA) supplemented with penicillin (100 IU/mL), streptomycin (100 μg/mL) (Gibco, NY, USA) and 2 mM L-glutamine (Paneco, Moscow, Russia), and incubated in a humidified atmosphere with 5% CO_2_ at 37 °C. The incubation medium was refreshed every 3–4 days to remove nonadherent cells. MSC at the third passage were used in the experiments. Viable cells were counted by staining with trypan blue and propidium iodide; cells with viability of >90% were used for culturing.

### 2.3. Traumatic Brain Injury Model

For all surgical procedures, rats were anesthetized with an intraperitoneal (i/p) injection of 300 mg/kg chloral hydrate. Additionally, to ensure proper pain relief in the perioperative and postoperative periods, we used repeated topical application of a long-acting local anesthetic of bupivacaine ointment. A feedback-controlled heating pad maintained the core temperature (37.0 ± 0.5 °C) during ischemia supplemented with an infrared lamp until awake.

In the present work, we employed our own modification of the earlier used model of focal open severe brain trauma in rats [19,20]. To perform the trauma, the left frontal part of the skull was trepanized above the sensorimotor cortex zone, and a movable Teflon piston 4 mm in diameter with depth of insertion of 2.5 mm was placed into it; this piston was struck from the height of 10 cm with a 50 g load sliding along a directing rail. For localization of the sensorimotor cortex zone, we used following stereotaxic coordinates; +1 to −3 mm anterior and posterior from bregma and +1.5 to +4.5 mm lateral from the midline. In sham-operated rats, the experiments were performed using the same protocol, except that trauma was excluded. The rats were injected i/v with a suspension of MSC (3 × 10^6^ cells/kg) and randomly divided into the following groups: (1) sham-operated rats injected with saline (Sham + saline, *n* = 6), (2) rats exposed to TBI and injection of saline (TBI + saline, *n* = 9), (3) rats exposed to TBI and injection with aMSC (TBI + aMSC, *n* = 8), (4) rats exposed to TBI and injection with pMSC (TBI + pMSC, *n* = 9), (5) rats exposed to TBI and injection with TBI + fMSC, *n* = 10). Brain damage was evaluated by analyzing brain T2 weighted MR-images obtained 14 days after the TBI, as described previously [21]. The neurological deficit was estimated by the limb-placing test, consisting of seven tasks, to assess forelimb and hindlimb responses to tactile and proprioceptive stimulation [22]. The rats were habituated to handling and tested before the operation, and at the 1st, 2nd, 4th, 7th and 14th post-ischemic days. For each task, the following scores were used: 2 points, normal response; 1 point, delayed and/or incomplete response; 0 points, no response. The total score over seven tasks was evaluated. Asymmetry of forelimbs use was evaluated in the cylinder test during spontaneous exploration of the cylinder walls [23]. The tests were performed on day 14 after TBI modeling. The rat was placed into a transparent cylinder (30 cm height and 20 cm in diameter) and its movements were recorded over 5–8 min with a camcorder positioned above the cylinder. The independent use of the contra- and ipsilateral forelimbs and, simultaneously, of both limbs during cylinder wall exploration in a rear posture were counted manually. The performance of the contralateral forelimb and simultaneous use of forepaws were calculated as a percentage of the total performance (sum of the independent and simultaneous touches to both left and right forelimbs).

### 2.4. Estimation of Mitochondrial Activity, Level of Autophagy and Reactive Oxygen Species (ROS) Production

Quantitative measurement of mitochondrial content and mitochondrial membrane potential were performed by loading of MSC with 1 µM MitoTracker Green and MitoTracker Red (Invitrogen, Thermo Fisher Scientific, Waltham, MA, USA), correspondently. To evaluate autophagy-lysosomal system, MSC were loaded with 2 µM Cyto-ID (Enzo Life Sciences, New York, NY, USA) or 1 µM LysoTracker Green (Invitrogen, Thermo Fisher Scientific, Waltham, MA, USA). The levels of ROS were estimated by stained with 1 µM 2′,7′-Dichlorofluorescin diacetate (DCFDA) (Invitrogen, Thermo Fisher Scientific, Waltham, MA, USA). Incubation cells with the probe was conducted in DMEM/F12 medium without sodium bicarbonate for 30 min at 37 °C. Then, MSC were washed with phosphate-buffered saline (PBS) and dissociated in 0.05% Trypsin-EDTA (Paneco, Moscow, Russia). The fluorescence intensity was evaluated by flow cytometry using Cytomics FC500 (Beckman Coulter, Brea, CA, USA). Cyto-ID, MitoTracker Green, LysoTracker Green and DCF-mediated fluorescence were measured on the FL1 channel, while MitoTracker Red-mediated fluorescence was evaluated on the FL3 channel. Argon laser with λex = 488 nm was used to excite the fluorescence.

### 2.5. Senescence Cell Detection Assay

For evaluation of a senescence-associated beta-galactosidase, MSC were seeded on Petri dishes with 50,000 cells per dish, and cultivated in a standard cultivation medium for 5 days. The MSC were stained using a commercially available senescence associated (SA)-β Gal kit (Cell Signaling Technology, Danvers, MA, USA), in accordance with the manufacturer’s guidelines. Briefly, the medium was removed from the cultures, and each sample was washed with PBS. After this, fixative solution was added, followed by a wash with PBS. Then, staining solution was added, and the samples were left at 36 °C for a day. Images were taken using phase-contrast microscope (Axio Vert.A1 Zeiss, Jena, Germany), and areas of SA-β Gal positive staining were evaluated by Image J software.

### 2.6. Real-Time Cell Proliferation Monitoring

Analysis of cells growth kinetics was performed using an RTCA iCELLigence™ instrument (ACEA, San Diego, CA, USA). This method is based on using electrical impedance of cell-covered electrodes [24], and may be used in particular for studies of adhesive culture cells’ proliferation and death [25]. The iCELLigence RTCA instrument was placed in a humidified incubator at 37 °C and 5% CO_2_. pMSC and aMSC were seeded on 8-well plates with microelectrodes. In part of the experiments, hypoxia was performed by reducing the oxygen concentration to 1% 24 h after seeding the cells in the plate, which lasted 96 h; the cells were kept in a standard cultural medium in multi-gas incubator Galaxy 170R (Eppendorf/NewBrunswick, Hamburg, Germany). In experiments to inhibit glycolysis, 2-deoxy-d-glucose (2-DG) was added to the MSC 42 h after the cells were seeded in the plate after dilution in a cultural medium for MSC. Cell quantity was given as a cell index; cell growth rate was estimated in the time interval of 14–24 h for normoxia and 75–85 h for hypoxia.

### 2.7. MSC Metabolism Analysis

Rat’s pMSC and aMSC were plated at 10,000 cells/well in 100 μL basal medium to a Seahorse 8-well miniplate. The cells were allowed to attach and grow to obtain a monolayer at 37 °C under 5% CO_2_. The plates were checked for uniform spreading and cell confluency under the microscope before metabolic analysis. Growth medium was aspirated, washed and replaced with DMEM, containing 2 mM L-glutamine, 1 mM sodium pyruvate and 10 mM D-glucose. Basal oxygen consumption rate (OCR) and extracellular acidification rate (ECAR) were measured in the Seahorse XFp analyzer (Seahorse Biosciences, Billerica, MA, USA), according to manufacturer’s guidelines. Additional measurements were performed after injection of five compounds affecting bioenergetics: oligomycin (4.5 μM, Sigma-Aldrich, St. Louis, MO, USA), carbonyl cyanide *m*-chlorophenyl hydrazone (CCCP) (10 μM, Sigma-Aldrich), D-glucose 10 mM, 2-DG (50 mM, Sigma-Aldrich) and rotenone (2.5 μM, Sigma-Aldrich). Three measurements were performed after the addition of each compound (total measurement time 100 min). Data analysis was performed using XFp Wave 2.6.1.

### 2.8. Western Blotting

MSC were dissociated from flasks with 0.05% trypsin-EDTA (Paneco, Moscow, Russia), then precipitated by centrifugation at 400× *g* for 5 min, and the resulting pellet was resuspended in 100 µL of saline solution. The protein content in the sample was determined using a commercial kit based on bicinchoninic acid (Sigma-Aldrich, St. Louis, MO, USA). The suspension was mixed with a 4-fold sample buffer containing 0.125 M Tris-HCl (pH 6.8), 4% sodium dodecyl sulfate, 40% glycerol, 0.05% bromophenol blue and 10% 2-mercaptoethanol, boiled for 5 min, and then used for immunoblotting. The same amount of total protein was applied to each well in the gel.

Samples of cell lysates were loaded on 15% Tris-glycine polyacrylamide gels (10 μg protein per lane). After electrophoresis, gels were blotted onto polyvinylidene difluoride (PVDF) membranes (Amersham Pharmacia Biotech, Amersham, UK). Membranes were blocked with 5% blocking agent (Amersham Pharmacia Biotech, RPN2125V, UK) in TBS containing 0.05% Tween-20, and subsequently incubated with primary antibodies: anti-Beclin-1 rabbit 1:1000 (c/n 3495, Cell Signaling, Danvers, MA, USA), anti-PCNA 1:1000 rabbit (c/n 13110, Cell Signaling, USA), anti-LC3 A/B rabbit 1:1000 (c/n ab84936, Abcam, Cambridge, UK), anti-LAMP-1 rabbit 1:1000 (c/n ab24170, Abcam, UK) and anti-β-actin mouse 1:2000 (c/n A2228, Sigma, USA). β-actin was used as the loading control. Membranes were then incubated with secondary antibodies: anti-rabbit IgG or anti-mouse IgG conjugated with horseradish peroxidase 1:5000–1:10,000 (Imtek, Moscow, Russia) and probed with Advansta Western Bright ECL kit (Advansta, San Jose, CA, USA). Detection was performed by V3 Western Blot Imager (BioRad, Hercules, CA, USA). Protein concentration was measured by bicinchoninic acid assay (Sigma, USA).

### 2.9. Transmission Electron Microscopy

MSC cultured in complete media were detached with 0.05% Trypsin-EDTA at 37 °C and centrifuged at 300× *g*. The supernatant was removed and the cell pellet was transferred to an Eppendorf vial. The cell pellet was centrifuged again, supernatant was removed and cells were fixed with 2.5% glutaraldehyde (Sigma, USA) on the Sorensen phosphate buffer (pH 7.4). for 2 h at 4 °C. Then, the cell pellet was washed in 0.1 M Na phosphate buffer. Fixed samples were stained with 1% OsO_4_ in PBS, followed by dehydration in ascending acetone concentrations, stained with 1% uranyl acetate in 70% acetone during dehydration and embedded in EPON™–Araldite mixture resin. After polymerization, 80 nm thick sections were made using the LKB Nova ultramicrotome (Stockholm, Sweden). Sections were collected on a carbon-coated Cu grid, stained with lead citrate according to Reynolds [26] and viewed with the electron microscope JEM-1400 (“JEOL”, Tokyo, Japan) at a 100 kV accelerating voltage.

### 2.10. Statistics

Statistical analyses were performed using Statistica 7.0 for Windows (StatSoft, Inc., Palo Alto, CA, USA). Values are given as mean ± standard error of the mean (SEM). Variance homogeneity was assessed with Levene’s test. Statistical differences in infarct volume between the groups were analyzed using one-way ANOVA with Tukey’s post hoc test. Statistical differences in the limb-placing and cylinder tests scores between the groups were analyzed using the Kruskal–Wallis test with the Mann–Whitney U-test (the Bonferroni post hoc correction was applied). Differences were considered significant at *p* ≤ 0.05. If not specified, biological samples and number of animals were used at least in triplicate.

## 3. Results

### 3.1. Effect of MSC of Different Age on Traumatic Brain Injury

**Figure 1:**
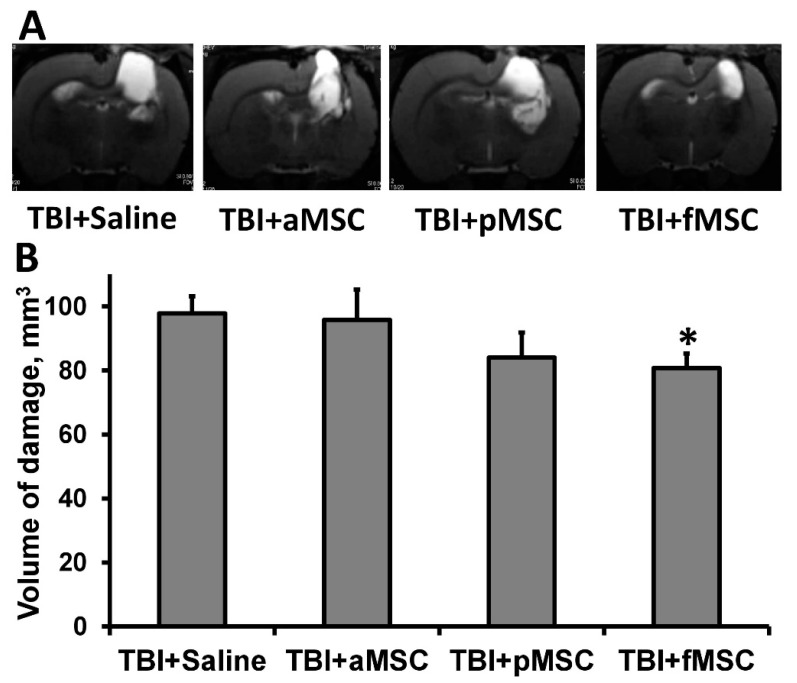
Effect of MSC transplantation on the volume of lesion in a rat traumatic brain: (**A**) Representative T2-weighted MR-images from coronal brain sections (0.8 mm thick, from rostral (top) towards caudal (bottom)), obtained on the 14th day after TBI. Light regions refer to ischemic areas after TBI. (**B**) Damage volume evaluated by MRI with analysis of T2-weighted images. * Denotes significant difference from the TBI + saline (*p* < 0.05) (One-way ANOVA, followed by Tukey’s post hoc analysis).

TBI caused extensive damage to the sensorimotor cortex, as shown by MR-images obtained on postoperative day 14 (Figure 1A). The volume of brain damage in the saline-injected (considered as control group) group was 97.8 ± 5.3 mm^3^. On the other hand, injection of fMSC significantly reduced brain damage volume by 18% (*p* < 0.05, Figure 1) when administered intravenously one day after TBI. In animals treated with pMSC, only a trend was observed towards a decrease in lesion volume, accounting for 83.9 ± 7.8 mm^3^, while the transplantation of aMSC did not affect lesions (Figure 1).

**Figure 2:**
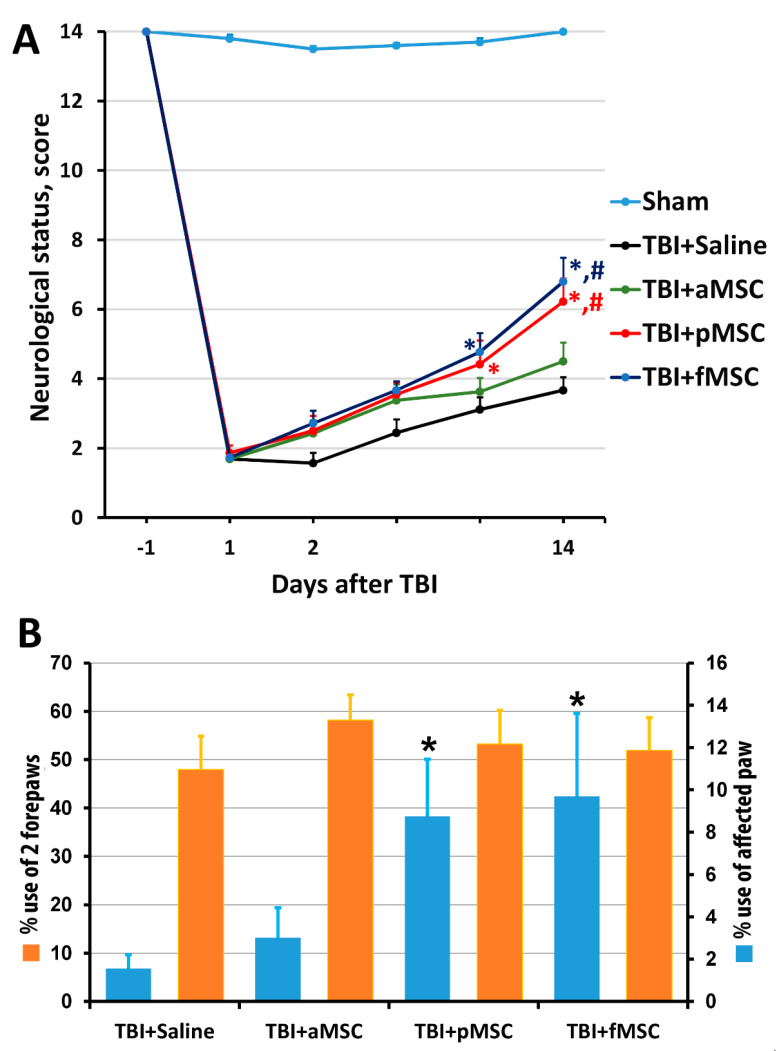
Effect of MSC on the neurological deficit of animals: (**A**) Neurological status scores estimated in the limb-placing test 1 day before and days 1, 2, 4, 7 and 14 after TBI. * *p* < 0.05 denotes significant difference from TBI + saline group; # *p* < 0.05 denotes significant difference from TBI + aMSC group (two-way ANOVA). (**B**) TBI induced limb asymmetry, measured by the cylinder test 14 days after the surgery. * *p* < 0.05 denotes a significant difference from TBI + saline group (Kruskal–Wallis test with the Mann–Whitney). Data are expressed as mean ± SEM.

The results of the limb-placing test revealed the development of a post-traumatic functional deficit in the right limbs (contralateral side to damaged cortex), whereas the left limbs stayed fully functional. Before the induction of trauma, the intact rats scored 14.0  ±  0 in the limb-placing test, while, on the first day after TBI, rats scored only 1.9  ±  0.2 in all groups. The transplantation of fMSC and pMSC improved neurological status, starting from the 7th day after TBI induction compared with the saline-treated group, and from the 14th day compared with TBI + aMSC group (Figure 2A). There were no significant differences between the scores in the TBI + saline and TBI + aMSC groups during all time points.

Using the cylinder test, we found that TBI results in an asymmetry in the use of the forelimbs. Normally, rats use both the left and right limbs in the same proportion when examining the walls of the closed space in the glass cylinder. Quantitative analysis showed that, before TBI modeling, the animals used the right forelimb and both forelimbs simultaneously, with a frequency of 21% and 56%, correspondingly (Figure 2B). Damage in the sensorimotor area of the right hemisphere caused a decrease in the use of the contralateral limb (left limb) to study the cylinder walls to 1.6% on the 14th day after TBI (Figure 2B). The transplantation of pMSC and fMSC partially restored the contralateral paw use to 8.7% and 9.7%, correspondingly (Figure 2B, *p* < 0.05), but not for the TBI + aMSC group.

One can notice that there is no correlation between the effect of administration of MSC on the brain structure and its physiological function: while the effect of MSC on lesion volume was minimal, functional recovery was remarkable, allowing us to discriminate the effects of MSC of different ages.

Since the fetal and perinatal MSC had about equal neuroprotective efficacy, in further study, only pMSC and aMSC were used in experiments.

### 3.2. Functional Impairment of MSC Caused by the Donor’s Age

**Figure 3:**
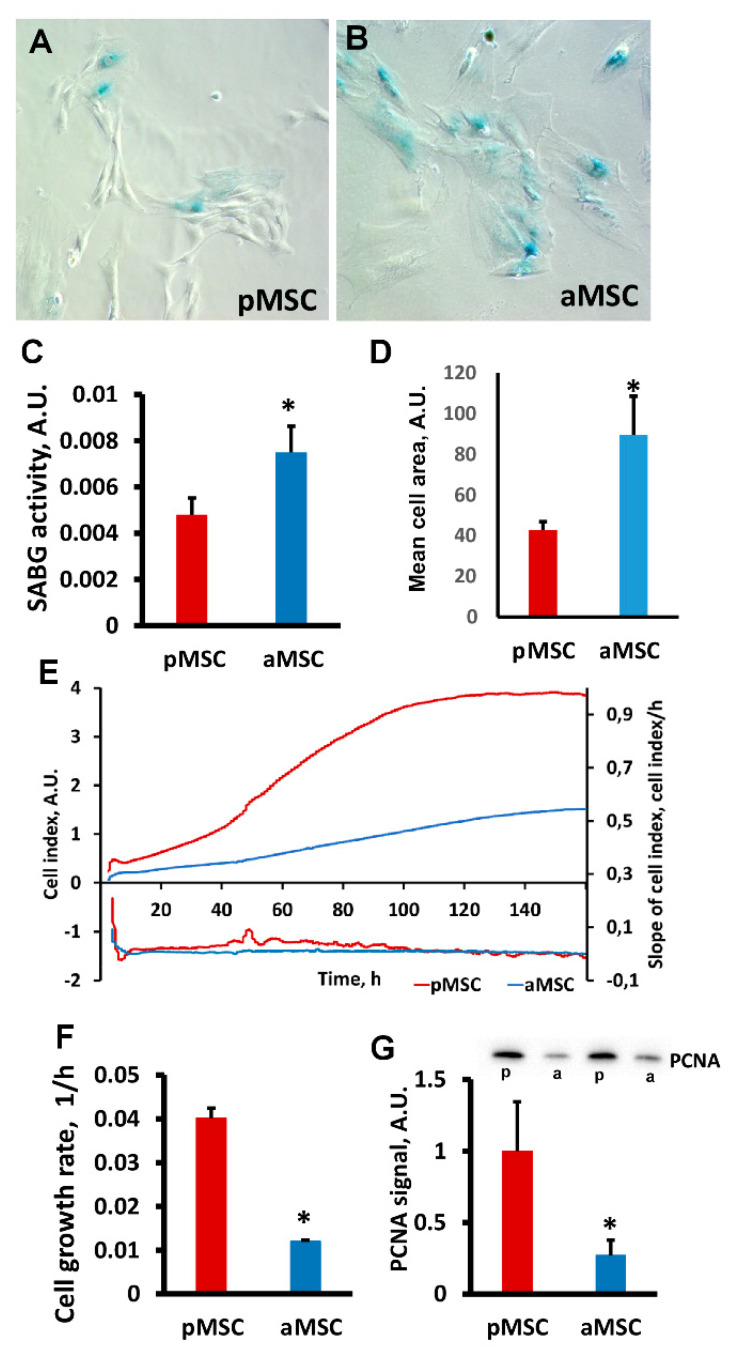
Estimation of proliferative potential and senescence of mesenchymal stromal cells, obtained from perinatal and old rats’ bone marrow. Representative images of SA-β-gal activity staining in pMSC (**A**) and aMSC (**B**). Senescent cells are stained blue. (**C**) Evaluation of β-galactosidase activity (SABG) normalized on cell area in pMSC and aMSC. (**D**) Mean cell area from donors of different ages. (**E**) Real-time curve of cell index and (**F**) the cell growth rate measured with the RTCA iCELLigence over 160 h. (**G**) Immunoblots for proliferating cell nuclear antigen (PCNA) in MSC and densitometry analysis of protein bands (diagram), respectively. P, perinatal, a, adult MSC. Actin staining was used as a loading control. Number of individual cultures for Western blot was 3. *: *p* < 0.05, (aMSC vs. pMSC).

A decrease in the neuroprotective properties of aMSC was associated with a significantly higher activity of cell senescence-associated β-galactosidase (SA-β gal) compared to pMSC. Visually (Figure 3A,B), we observed a more pronounced specific SA-β gal-positive staining in MSC obtained from old rats. Digital analysis of the obtained microscopic images confirmed that the area of the blue positive color, which characterizes the presence of active SA-β gal normalized by the total cell area, is higher in old MSC (Figure 3C, *p* < 0.05). Significant changes in cell size between groups were also recorded, indicating a large area occupied by old MSC (Figure 3D), which is a characteristic feature of a senescent culture.

The proliferation rate of pMSC and aMSC was estimated by real-time monitoring of the cell index using an iCELLigence device. We found that pMSC characterized by a more rapid proliferation exceeded the growth of aMSC by more than 4-fold (Figure 3E,F). An analysis of growth rate measured as a slope of the cell index increment proved a higher rate of pMSC during the exponential phase of cell growth (Figure 3F). The levels of proliferating cell nuclear antigen (PCNA), a well-known proliferation marker, were three times lower in aMSC compared with the pMSC using Western blotting (Figure 3G). Thus, the age of the MSC donor affects the proliferative ability of MSC and signs of senescence.

### 3.3. Evaluation of MSC Proliferation under Hypoxia and Glycolysis Inhibition

**Figure 4:**
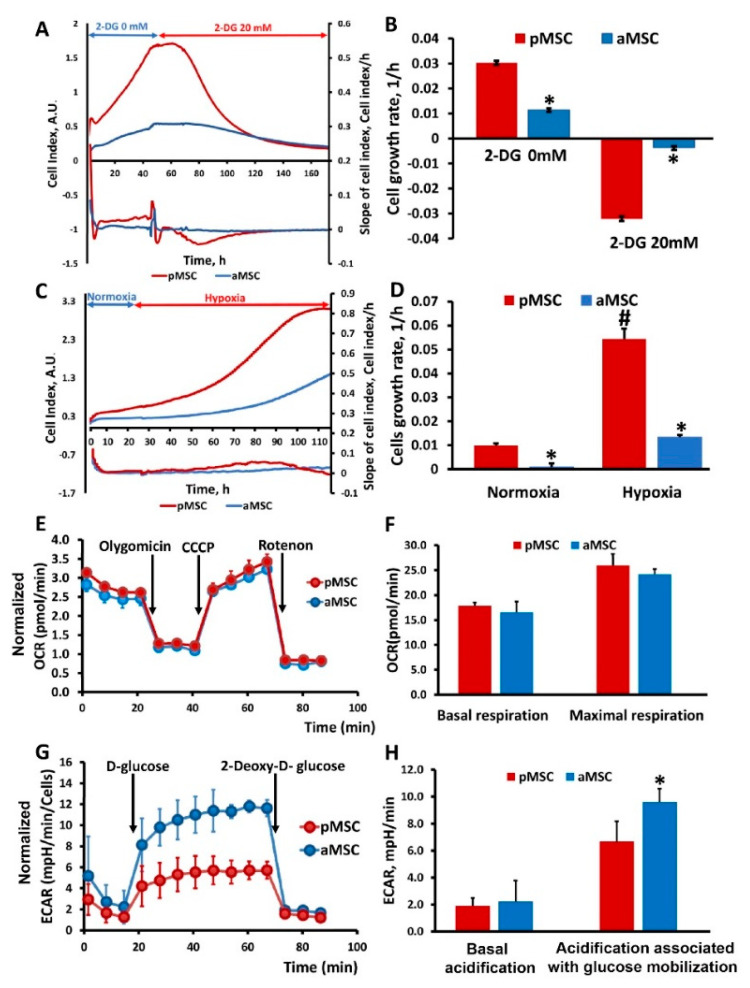
Glucose utilization and mitochondrial respiratory function in perinatal and adult MSC. Real-time proliferation and growth rate of MSC obtained from neonatal and adult rats under conditions of glycolysis inhibition with 20 mM 2-Deoxy-D-glucose (2-DG) (**A**) and during hypoxia (**C**). Cell growth rate (slope of cell index) measured after 2-DG treatment (**B**) and in normoxia/hypoxia conditions (**D**). (**E**) Representative oxygen consumption traces and (**F**) basal and maximal oxygen consumption rate (OCR) analysis in MSC in response to oligomycin (4.5 µM), CCCP (10 µM) and rotenone (2.5 µM). (**G**) Time course of extracellular acidification rate (ECAR), at least partially reflecting glycolytic function in MSC. Kinetics of extracellular acidification rate (ECAR) in pMSC and aMSC cultures in response to glucose (10 mM) and 2-DG (50 mM) treatment. (**H**) Calculated non-glycolytic acidification and glycolytic capacity in MSC. * *p* < 0.05 denotes a significant difference between the “pMSC” and “aMSC”; # *p* < 0.05 denotes a significant difference between the normoxia and hypoxia conditions for pMSC.

To study the effect of inhibition of the main methods of energy production in the cell on the proliferation of MSC of different ages, changes in the cell index were analyzed in real-time when cultured in the presence in a medium of 2-DG, a competitive inhibitor of hexokinase (an enzyme that catalyzes the first reaction of glycolysis), ultimately retarding glycolysis (Figure 4A,B), and under hypoxic conditions inhibiting respiration (Figure 4C,D). Cell growth rates were calculated from the recorded curves of changes in the cell index. Cultivation of MSC in the presence of 20 mM 2-DG led to a sharp drop in the growth rate in the case of pMSC, which was accompanied by a cease of cell proliferation, and further visual analysis revealed cell death. A similar effect was observed in the aMSC culture but, given that their growth rate before the addition of 2-DG was lower, the drop in growth rate during incubation with 2-DG was less pronounced compared to pMSC (Figure 4B). An analysis of the growth rate based on the values of the cell index under hypoxia conditions shows that, in aMSC, it increases by more than 10 times, while, in pMSC, it increases by five times compared to the rate calculated under normoxic conditions (Figure 4D).

### 3.4. Assessment of Energy Metabolism in MSC of Different Ages of the Donor

To assess the energy metabolism of MSC isolated from animals of different ages, oxygen consumption rate (OCR) was measured. Each curve in Figure 4E characterizes cell respiration, where each point corresponds to the averaged values of three independent cultures for each of the MSC types being compared. Statistically significant differences in OCR between aMSC and pMSC were not observed for almost all the studied exposures, including baseline respiration rate (basal respiration), effects of oligomycin (an ATP synthase inhibitor), CCCP and rotenone/antimycin (inhibitors of the NADH dehydrogenase complex and the cytochrome-bc1 complex of the mitochondrial respiratory chain that, correspondingly, completely inhibit oxygen consumption by mitochondria). Basal and maximal respiration were also calculated on the basis of OCR (Figure 4F). However, there were no statistically significant differences in these parameters between the pMSC and aMSC groups.

Secondly, we measured the extracellular acidification rate (ECAR) of the MSC, which is an indirect readout of L-lactate production after the sequential injection of modulators of the aerobic glycolysis (D-glucose, oligomycin, 2-DG). As it can be observed in Figure 4G, basal ECARs of pMSC and aMSC did not differ from each other. Next, we added D-glucose (10 mM final concentration) to evaluate cells’ ability to upregulate glycolysis when glucose is available. After the glucose supplementation, aMSC had higher levels of ECAR compared to pMSC (Figure 4G, 4 to 11 measurements), indicating an increased capacity for glucose utilization. Numerically, the increase in ECAR in aMSC after D-glucose addition was 20% vs. pMSC (Figure 4H), which may indicate a greater activity of glycolysis as an energy source in aMSC. After the addition of 2-DG, glycolysis was inhibited, which was expressed as a significant decrease in ECAR in both groups. At the same time, the rate of acidification fell almost to the values of the first stage, when there was no glucose in the medium. The glucose-dependent increase of the ECAR, as well as its inhibition by 2-DG, may suggest that the observed acidification has a glycolytic origin.

### 3.5. Evaluation of the Autophagy Activity, the Level of ROS and the Mitochondrial Membrane Potential in the MSC of Perinatal and Adult Rats

**Figure 5:**
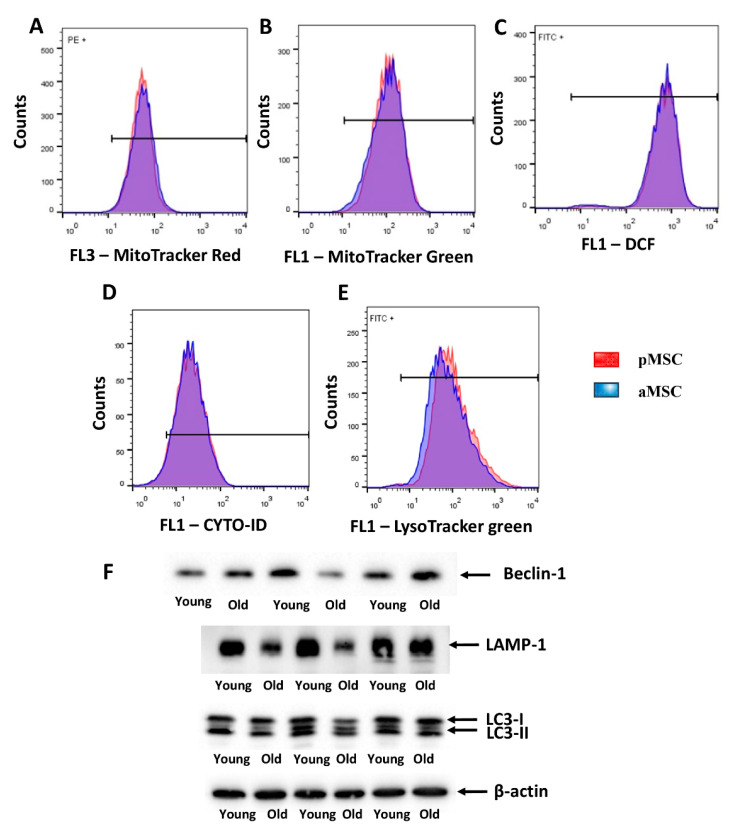
Evaluation of the mitochondrial potential, the autophagic activity and the levels of ROS in the perinatal and adult MSC obtained from the femoral bones. Flow cytometry of primary cultures of MSC loaded with MitoTracker Red (**A**) and Green (**B**) for the estimation of mitochondrial transmembrane potential and mitochondria content, correspondently. (**C**) ROS production as measured by DCF fluorescence in pMSC and aMSC. (**D**) Cyto-ID assay and (**E**) LysoTracker were used to evaluate the activity of autophagy and lysosomes in MSC using flow cytometry. (**F**) The autophagy markers levels of Beclin-1, LAMP-1 and LC-3-I/II, in pMSC and aMSC. A total of 50,000 cells and 3 samples were used for flow cytometry and Western blot analysis correspondingly.

Mitochondrial membrane potential was estimated by flow cytometry using MitoTracker™ Red FM, which accumulates in mitochondria in a membrane potential mode. MitoTracker™ Green FM was used for the evaluation of mitochondrial content in MSC. We did not reveal differences in both mitochondrial transmembrane potential and mitochondrial mass between pMSC and aMSC (Figure 5A,B). Additionally, we evaluated the level of oxidative stress by measuring, in MSC, the fluorescence of a ROS-sensitive probe, DCF. A population-based analysis of MSC did not reveal any differences in fluorescence of the DCF in cells obtained from aged rats and pMSC (Figure 5C). In order to assess the autophagic in aging MSC, the level of autophagosomes was evaluated by using Cyto-ID, which selectively accumulated in autophagosomes and autophagolysosomes, and specifically lysosomes, using LysoTracker green, correspondently. MSC from old rats did not show any differences in fluorescence intensity of these probes when compared with pMSC (Figure 5D,E). Analysis of protein markers of autophagy, such as Beclin-1, LAMP-1 and LC3 II/LC3 I ratio, revealed only a decrease in the content of LAMP-1 in aMSC (Figure 5F), while the other parameters did not change with aging.

### 3.6. Aging of MSC Impacts Ultrastructure

**Figure 6:**
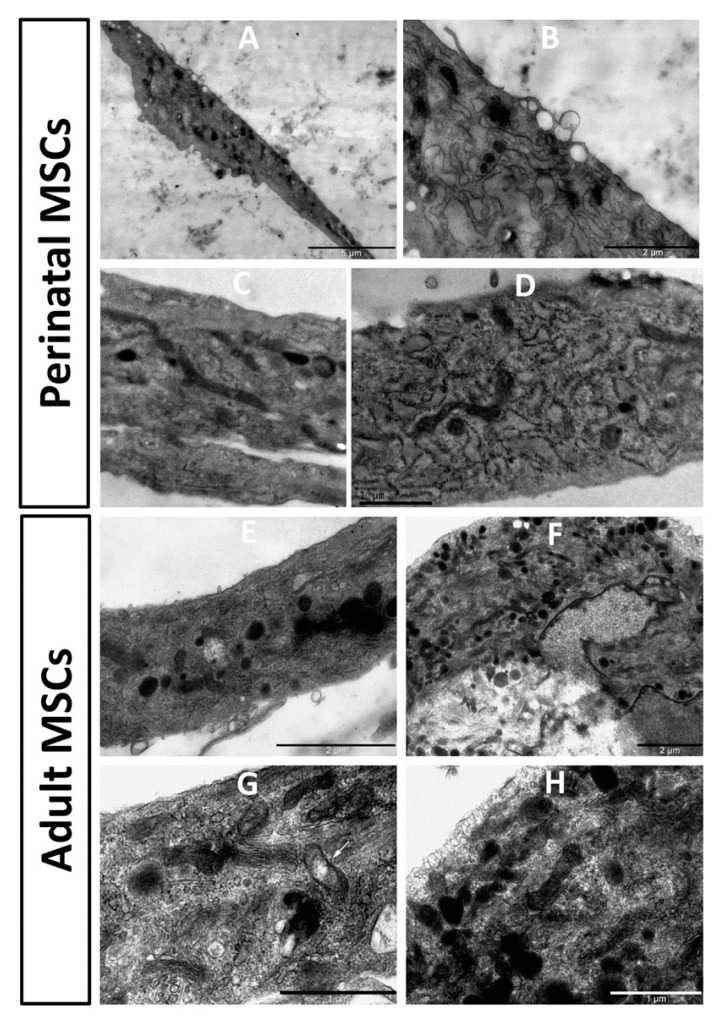
Electron microscopic images of the perinatal (**A**–**D**) and adult (**E**–**H**) MSC from rat bone marrow. Vesicular structures on the cell border are visible in (**A**), with a high zoom of the same cell in (**B**). pMSC contain mitochondria as dark extended profiles existing in the orthodox configuration, while mitochondria in cells from adult MSC preferentially had round profiles, demonstrating partial fragmentation of mitochondrial reticulum. Additionally, slight local (shown by an arrow in (**G**)) and general (shown by arrowhead in (**G**)) swelling of intracristae space and matrix in these mitochondria are obvious.

Ultrastructure of stem cells obtained from young and old rats showed noticeable differences (Figure 6). In pMSC, mitochondria were mostly represented by extended structures, with a predominant condensed matrix over the entire mitochondrial population, which could indicate high respiratory activity and low levels of mitochondrial heterogeneity. The endoplasmic reticulum was clearly expressed with characteristic and abundant polysomes, indicating active protein synthesis in cells. A number of multilamelar structures were observed, apparently representing autophagosomal/lipofuscinic formations as products of degradation of intracellular elements, primarily mitochondria. Multiple structures, apparently preceding exosome generation, were observed on the plasma membrane.

In cells obtained from old rats, the endoplasmic reticulum was not as clearly expressed as in young cells, with low levels of polysomes, which could indicate low protein synthetic activity. In the mitochondrial population, a significant part was represented by more round structures, which is evidence of an increased share of fragmented mitochondria. This sign could indicate an increase in mitochondrial fission and/or an impairment of the process of mitochondrial fusion. The ultrastructure of mitochondria was equally represented by both condensed and orthodox mitochondria, with frequent local enlightenments in the matrix, which indicated an increase in the heterogeneity of the mitochondrial population, and therefore their different respiratory activity and energetics. Autophagosomal structures were rare.

## 4. Discussion

When considering the problems of aging of the biological system, the examples of accelerated aging caused by genetic and epigenetic factors are often presented with mitochondria playing a key role [27,28,29,30]. However, it seems appropriate to talk about programmed delayed aging, the subject of which is as relevant as the problem of accelerated aging. One example of the intrinsic anti-aging mechanism, which includes the asymmetric division ([31], reviewed in [4]) is characteristic of budding yeast cells (so called replicative aging [32]), where the best quality mitochondria are given to the bud [33] while the mother cell does not receive the best mitochondria. This ultimately sets unequal initial conditions for the development of the aging process, in which the daughter cell, by definition, must senesce at a lower rate than the mother cell. The reverse pattern is observed for stem cells that also perform asymmetric division, in which the best mitochondria are transferred to the cell that remains in the stem cell pool (and this determines the stemness [3]), while another division product of the worst quality undergoes a differentiation process to replace the lost somatic cell. If such a mechanism of banned or delayed senescence does exist, stem cells undergoing such a life mode characterized by anti- or retarded senescence mechanisms in extreme cases could potentially be able to be expressed as intrinsically immortal phenotypes.

On the other hand, there are data arguing with such hypothesis and presenting evidence that stem cells undergo time-dependent changes in their structure and functions identical to somatic cells [11], e.g., changes in the pattern of gene expression in the same tissue at different ages [14,15]. Using the model of traumatic brain injury, the data of this study demonstrate that the therapeutic potential of MSC isolated from rats of different age fades away proportionally to the rise in the donor’s age. Although we did not characterize the MSC taken from animals of different ages, we were based on the data obtained in the study [12], where such a characterization was carried out by excluding the group of very old animals which we used in our work. Their study indicated, for the first time, that MSC have significant differences in their proliferative, pluripotency and metabolism profiles, and those differences are age dependent. However, the characterization of populations of MSC from different groups sorted by age and flow cytometry revealed no statistically significant differences on the level of mesenchymal markers (all groups contained 30 ± 5% of positive cells for CD29 and 75 ± 5% of positive cells for CD90). On the other hand, the bioenergetic mode contributes significantly to this chronological decay, since mitochondrial energetics supported by respiration seem to stay almost identical in MSC from young and old animals, while young cells acquire more intensive glucose utilization, which possibly provides a higher flexibility to cope with pathological challenges. Higher glucose utilization flux in young MSC supports higher cell growth; moreover, when working in this mode, cells become less dependent on an oxygen supply, resulting in them acquiring a higher tolerance to hypoxia and mitochondrial substrate delivery. The novelty of this study is that it demonstrates the association of changes in the therapeutic ability of stem cells with changes in the structure of mitochondria and entire bioenergetics. The limitation of this study is that cells isolated from their donor organism were used, followed by cultivation of up to three passages. We assume that the cultivation process does not introduce critical changes compared to initial cells in vivo, but the necessity of cultivation is dictated by the clinical needs for sufficient administrated material to achieve therapeutic effects.

Similar results have been recently obtained, with a rather interesting interpretation of the Seahorse data [34]. The authors sought to understand the nature of the immune-suppressing properties of the MSC when studying changes in the metabolism of MSC after their priming with proinflammatory cytokines, tumor necrosis factor-α (TNF-α) and interferon-γ (IFN-γ). Although the mitochondrial energetics of MSC did not show significant changes after priming, extracellular acidification rate, which is conventionally interpreted as a direct reflection of glycolytic activity, was significantly enhanced, being fully blocked by a competitive inhibitor of hexokinase, 2-deoxyglucose. Thus, the authors concluded that priming switches the MSC energetics toward enhanced glycolysis. In addition, they found that primed MSC demonstrate enhanced modification of a signal transducer and activator of transcription (STAT1) by O-linked *N*-acetylglucosamine (*O*-GlcNAcylation) and *N*-glycosylation, mediated via bifurcation of a glycolytic pathway after Fructose 6-P formation. If this is the case, literally speaking, redirection of Fructose 6-P associated with enhanced glucose utilization cannot be interpreted as enhanced glycolysis since, by definition, glycolysis is the conversion of glucose to lactic acid (anaerobic glycolysis) or to acetylCoA from glycolytic pyruvate (aerobic glycolysis). Whether inflammatory priming was associated with conventionally defined glycolysis has not been clarified which, theoretically, could be performed by using glycolytic inhibitors downstream of a bifurcation point (after Fructose 6-P), but we cannot rule out that utilization of glucose causes both the activation of glycolysis and an enhanced flow of the hexamine pathway. The attractive mechanism suggested by the authors is at least an indication that the utilization of glucose can perform both an energetic (via glycolysis) and a signaling role (note that, while the former yields more ATP, the latter is exclusively an ATP-consuming process). Ultimately, in the authors’ model, a signaling function associated with higher glucose utilization triggers the expression of STAT1-dependent indoleamine 2,3 dioxygenase (IDO), which is itself an anti-inflammatory trigger. We speculate that a similar conclusion can be drawn considering data from this study demonstrating higher glucose utilization in MSC from young animals, possibly due to enhanced signaling and without excluding higher glycolytic flux.

However, regardless of mitochondrial energetics, there were obvious changes in mitochondrial ultrastructure, indicating some regressive alterations. Conventionally, mitochondrial ultrastructure is subdivided into a number of ultrastructural substrates, the two main ones being the orthodox and condensed matrix configuration [35,36,37]. Condensed configuration corresponds to a state with diminished membrane potential, either due to intrinsic uncoupling or to the transition from State 4 (controlled or minimal respiration) to State 3 (almost maximally active respiration caused by ATP synthesis). Thus, it is not easy to attribute the ultrastructure of mitochondria to their quality, but we can assume that the predominant condensed state of the mitochondrial matrix in cells taken from young animals may correspond to a more active turnover of ATP, which leads to a higher activity of ATP synthase associated with active proliferative capacity. Earlier, we underwent the analysis of mitochondrial ultrastructure in tubular renal cells, confirming the partial rejuvenating effect of diet restriction [38]. While the mitochondria in MSC taken from young animals had a condensed configuration, in cells from old rats, the configuration shifted to a more orthodox state, which we interpreted as a transition to a state of mitochondria with lower membrane potential. Data from direct measurements of the membrane potential using a membrane potential-dependent probe confirmed this conclusion. However, importantly, while the predominant configuration of MSC mitochondria taken from young animals showed a condensed state, the mitochondria of cells taken from old animals had both a condensed and orthodox configuration showing increased mitochondrial heterogeneity, which is detrimental. In addition, the three-dimensional structure of the mitochondrial reticulum is also subject to change in age towards a more fragmented state, which we associate with high levels of oxidative stress [39].

These morphological changes may be even stay apart from pure mitochondrial energetics but belong to alternative mitochondrial functions which determine synthetic, regulatory, adaptive and other functions [40], and which may greatly contribute to both accelerated and delayed aging. However, we must admit that observed mitochondrial fragmentation in MSC indirectly points to at least mild oxidative stress [39,41], which may be associated with the aging of any biological system.

Despite the ambiguous interpretation of aging, most researchers point to mitochondria as a key element in this multimodal process. Oxidative stress of any origin, including a mitochondrial one, remains a basic element of the oxidative/free radical/mitochondrial theory of aging [42] which, to a significant extent, is applicable to the senescence of stem cells, with the mentioned limitations caused by their asymmetric division. The oxidative theory of aging deserves special attention, according to which aging is accompanied by increased levels in the cell of unwanted oxidized products of cell components: proteins, lipids, nucleic acids and small molecules. Such an evolutionary (relatively slow) increase can lead to rapid revolutionary intra-mitochondrial, intracellular, intra-organ and intra-organismal oxidative changes that lead to the death of the mitochondria, cell, organ or organism [43,44,45,46]. However, the data on the presence of such oxidized products in the organism of one of the longest living organisms, the naked mole-rat, at least outwardly compromise with the oxidative theory of aging [47], which stimulates the search of alternative or complementary arguments explaining time-dependent changes in a biological system. Apparently, the search must be widened by considering the synthetic and regulatory role of mitochondria, apart from their energetics. Intuitively, it seems reasonable to consider the management of cellular activity through the cross talk between mitochondrial and nuclear genomes, especially focusing on management from the mitochondrial side, or, as it was called, by retrograde signaling. Such regulatory age-determined signaling from mitochondrion to nucleus, lying at the nexus of metabolic regulation, has been neatly demonstrated for replicative aging in yeast [48]. Interestingly, an important role in retrograde signaling was ascribed to mitochondria-generated peptides [49].

More clearly, an understanding begins to develop regarding the fact that metabolic alterations accumulated over time are a “metabolic clock” which controls aging [50,51,52]. The key point which almost received consensus is that the balance between energy supply and demand is the requisite for a long and healthy life span [53]. Within these frames, reducing energy expenditure in its practical form, expressed as caloric restrictions, can potentially lead to a longer existence of the biological system, and at least slow down the aging process [54]. To some extent, the lower energy yield observed in this study in pMSC, due to the change in contribution of the glycolytic pathway to the total metabolism, is able to provide both adaptive healthy properties [38] and a longer life [55]. Ultimately, presumably acquired anti-senescence mechanisms in stem cells may provide the speed of aging at a rather low level, which requires further detailed investigation.

## Data Availability

The data presented in this study are available the article.
